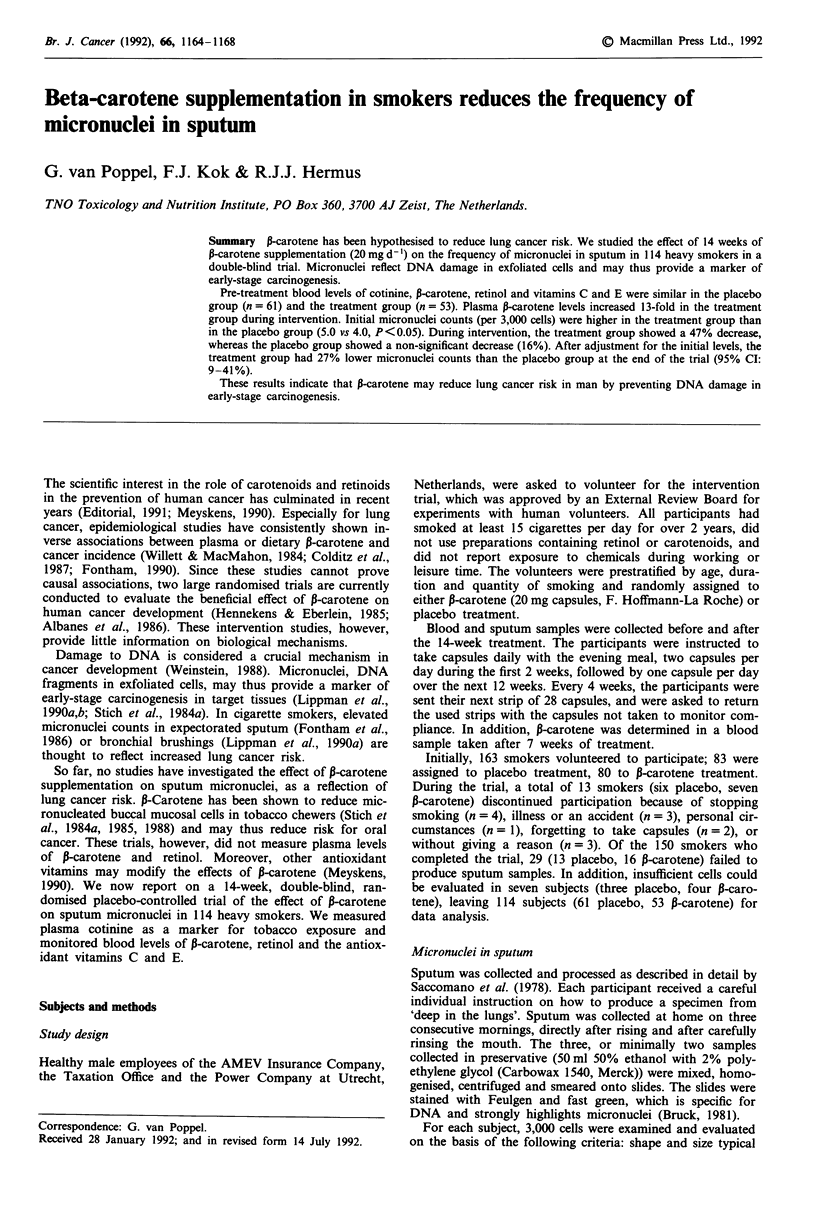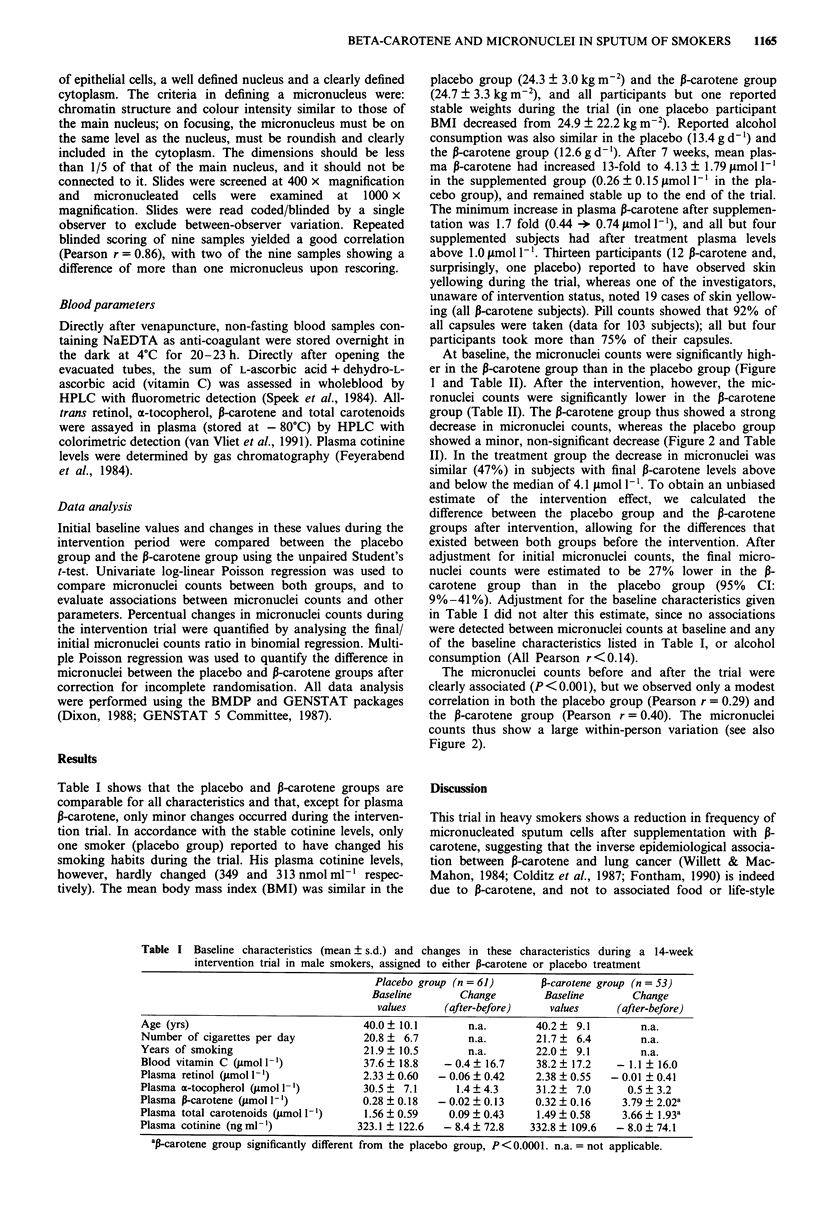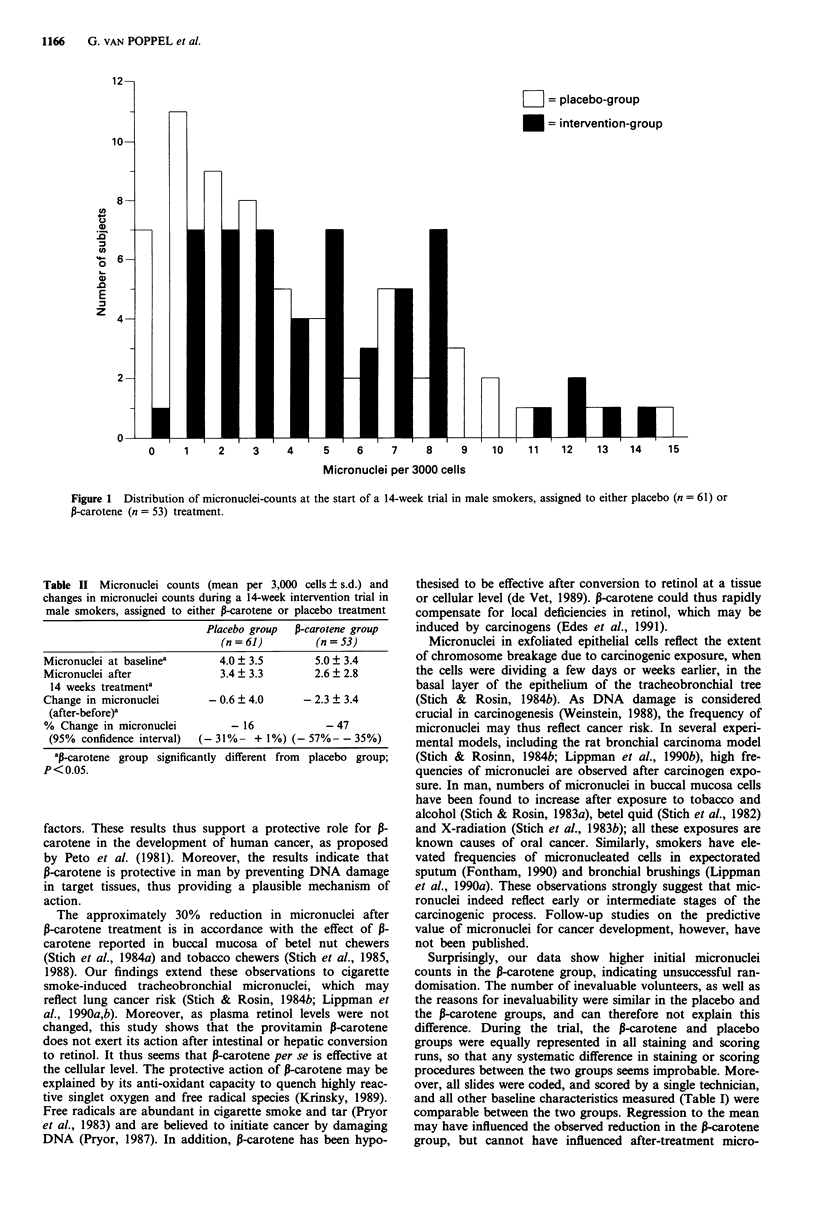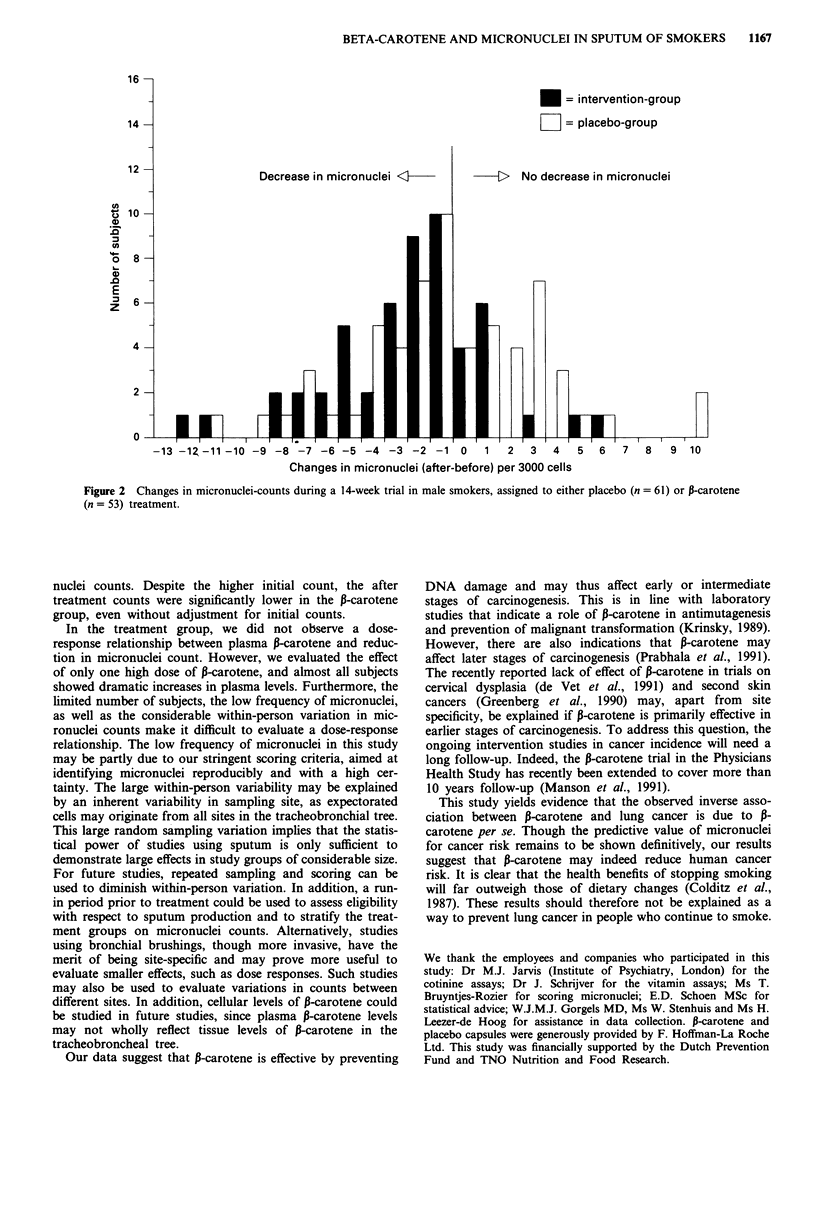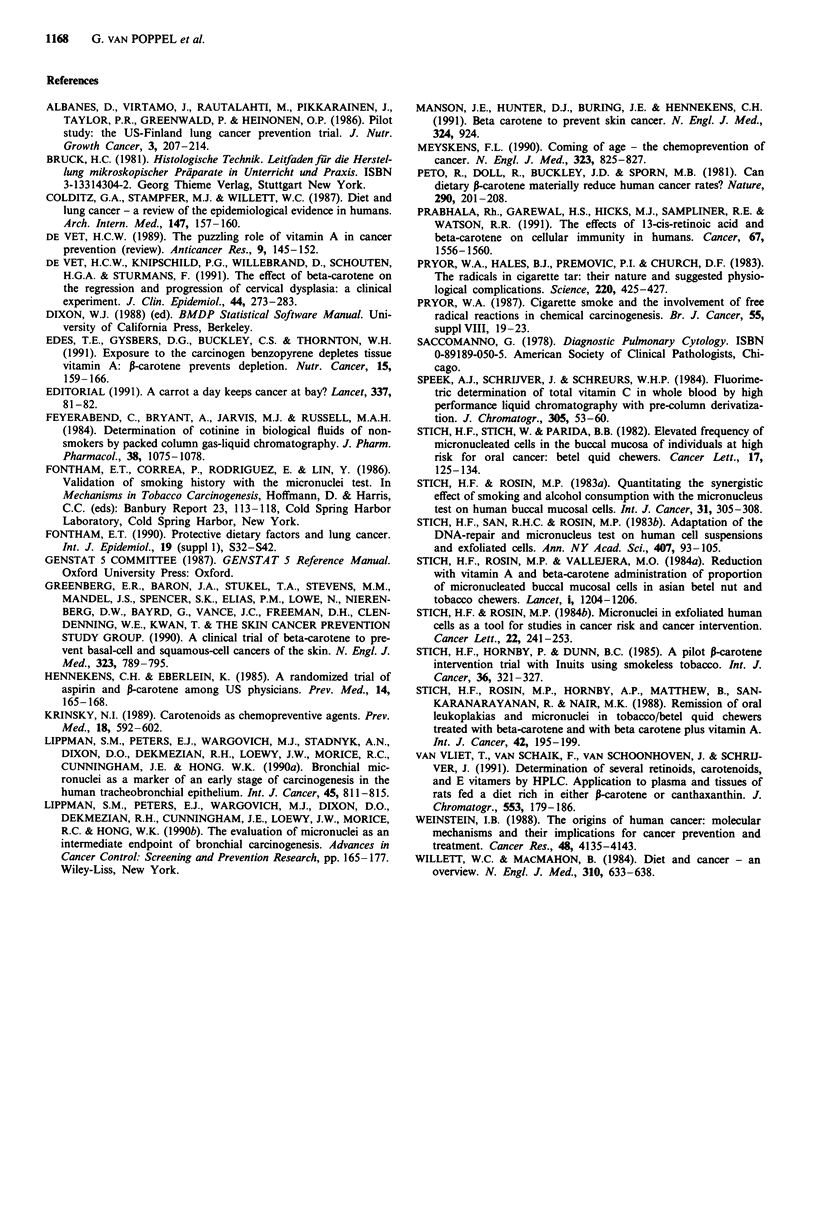# Beta-carotene supplementation in smokers reduces the frequency of micronuclei in sputum.

**DOI:** 10.1038/bjc.1992.428

**Published:** 1992-12

**Authors:** G. van Poppel, F. J. Kok, R. J. Hermus

**Affiliations:** TNO Toxicology and Nutrition Institute, Zeist, The Netherlands.

## Abstract

beta-carotene has been hypothesised to reduce lung cancer risk. We studied the effect of 14 weeks of beta-carotene supplementation (20 mg d-1) on the frequency of micronuclei in sputum in 114 heavy smokers in a double-blind trial. Micronuclei reflect DNA damage in exfoliated cells and may thus provide a marker of early-stage carcinogenesis. Pre-treatment blood levels of cotinine, beta-carotene, retinol and vitamins C and E were similar in the placebo group (n = 61) and the treatment group (n = 53). Plasma beta-carotene levels increased 13-fold in the treatment group during intervention. Initial micronuclei counts (per 3,000 cells) were higher in the treatment group than in the placebo group (5.0 vs 4.0, P < 0.05). During intervention, the treatment group showed a 47% decrease, whereas the placebo group showed a non-significant decrease (16%). After adjustment for the initial levels, the treatment group had 27% lower micronuclei counts than the placebo group at the end of the trial (95% CI: 9-41%). These results indicate that beta-carotene may reduce lung cancer risk in man by preventing DNA damage in early-stage carcinogenesis.


 © Macmillan Press Ltd., 1992

### Beta-carotene supplementation in smokers reduces the frequency of micronuclei in sputum

G. van Poppel, F.J. Kok & R.J.J. Hermus

*TNO Toxicology and Nutrition Institute, PO Box 360, 3700 AJ Zeist, The Netherlands.*

**Summary** β-carotene has been hypothesised to reduce lung cancer risk. We studied the effect of 14 weeks of β-carotene supplementation (20 mg $d^{-1}$) on the frequency of micronuclei in sputum in 114 heavy smokers in a double-blind trial. Micronuclei reflect DNA damage in exfoliated cells and may thus provide a marker of early-stage carcinogenesis.

Pre-treatment blood levels of cotinine, β-carotene, retinol and vitamins C and E were similar in the placebo group ($n = 61$) and the treatment group ($n = 53$). Plasma β-carotene levels increased 13-fold in the treatment group during intervention. Initial micronuclei counts (per 3,000 cells) were higher in the treatment group than in the placebo group (5.0 *vs* 4.0, $P < 0.05$). During intervention, the treatment group showed a 47% decrease, whereas the placebo group showed a non-significant decrease (16%). After adjustment for the initial levels, the treatment group had 27% lower micronuclei counts than the placebo group at the end of the trial (95% CI: 9-41%).

These results indicate that β-carotene may reduce lung cancer risk in man by preventing DNA damage in early-stage carcinogenesis.

The scientific interest in the role of carotenoids and retinoids in the prevention of human cancer has culminated in recent years (Editorial, 1991; Meyskens, 1990). Especially for lung cancer, epidemiological studies have consistently shown inverse associations between plasma or dietary β-carotene and cancer incidence (Willett & MacMahon, 1984; Colditz *et al.*, 1987; Fontham, 1990). Since these studies cannot prove causal associations, two large randomised trials are currently conducted to evaluate the beneficial effect of β-carotene on human cancer development (Hennekens & Eberlein, 1985; Albanes *et al.*, 1986). These intervention studies, however, provide little information on biological mechanisms.

Damage to DNA is considered a crucial mechanism in cancer development (Weinstein, 1988). Micronuclei, DNA fragments in exfoliated cells, may thus provide a marker of early-stage carcinogenesis in target tissues (Lippman *et al.*, 1990*a*,*b*; Stich *et al.*, 1984*a*). In cigarette smokers, elevated micronuclei counts in expectorated sputum (Fontham *et al.*, 1986) or bronchial brushings (Lippman *et al.*, 1990*a*) are thought to reflect increased lung cancer risk.

So far, no studies have investigated the effect of β-carotene supplementation on sputum micronuclei, as a reflection of lung cancer risk. β-Carotene has been shown to reduce micronucleated buccal mucosal cells in tobacco chewers (Stich *et al.*, 1984*a*, 1985, 1988) and may thus reduce risk for oral cancer. These trials, however, did not measure plasma levels of β-carotene and retinol. Moreover, other antioxidant vitamins may modify the effects of β-carotene (Meyskens, 1990). We now report on a 14-week, double-blind, randomised placebo-controlled trial of the effect of β-carotene on sputum micronuclei in 114 heavy smokers. We measured plasma cotinine as a marker for tobacco exposure and monitored blood levels of β-carotene, retinol and the antioxidant vitamins C and E.

### Subjects and methods

### *Study design*

Healthy male employees of the AMEV Insurance Company, the Taxation Office and the Power Company at Utrecht, Netherlands, were asked to volunteer for the intervention trial, which was approved by an External Review Board for experiments with human volunteers. All participants had smoked at least 15 cigarettes per day for over 2 years, did not use preparations containing retinol or carotenoids, and did not report exposure to chemicals during working or leisure time. The volunteers were prestratified by age, duration and quantity of smoking and randomly assigned to either β-carotene (20 mg capsules, F. Hoffmann-La Roche) or placebo treatment.

Blood and sputum samples were collected before and after the 14-week treatment. The participants were instructed to take capsules daily with the evening meal, two capsules per day during the first 2 weeks, followed by one capsule per day over the next 12 weeks. Every 4 weeks, the participants were sent their next strip of 28 capsules, and were asked to return the used strips with the capsules not taken to monitor compliance. In addition, β-carotene was determined in a blood sample taken after 7 weeks of treatment.

Initially, 163 smokers volunteered to participate; 83 were assigned to placebo treatment, 80 to β-carotene treatment. During the trial, a total of 13 smokers (six placebo, seven β-carotene) discontinued participation because of stopping smoking ($n = 4$), illness or an accident ($n = 3$), personal circumstances ($n = 1$), forgetting to take capsules ($n = 2$), or without giving a reason ($n = 3$). Of the 150 smokers who completed the trial, 29 (13 placebo, 16 β-carotene) failed to produce sputum samples. In addition, insufficient cells could be evaluated in seven subjects (three placebo, four β-carotene), leaving 114 subjects (61 placebo, 53 β-carotene) for data analysis.

### *Micronuclei in sputum*

Sputum was collected and processed as described in detail by Saccomano *et al.* (1978). Each participant received a careful individual instruction on how to produce a specimen from 'deep in the lungs'. Sputum was collected at home on three consecutive mornings, directly after rising and after carefully rinsing the mouth. The three, or minimally two samples collected in preservative (50 ml 50% ethanol with 2% polyethylene glycol (Carbowax 1540, Merck)) were mixed, homogenised, centrifuged and smeared onto slides. The slides were stained with Feulgen and fast green, which is specific for DNA and strongly highlights micronuclei (Bruck, 1981).

For each subject, 3,000 cells were examined and evaluated on the basis of the following criteria: shape and size typical

Correspondence: G. van Poppel.
Received 28 January 1992; and in revised form 14 July 1992.

of epithelial cells, a well defined nucleus and a clearly defined cytoplasm. The criteria in defining a micronucleus were: chromatin structure and colour intensity similar to those of the main nucleus; on focusing, the micronucleus must be on the same level as the nucleus, must be roundish and clearly included in the cytoplasm. The dimensions should be less than 1/5 of that of the main nucleus, and it should not be connected to it. Slides were screened at 400 × magnification and micronucleated cells were examined at 1000 × magnification. Slides were read coded/blinded by a single observer to exclude between-observer variation. Repeated blinded scoring of nine samples yielded a good correlation (Pearson $r = 0.86$), with two of the nine samples showing a difference of more than one micronucleus upon rescoring.

### *Blood parameters*

Directly after venapuncture, non-fasting blood samples containing NaEDTA as anti-coagulant were stored overnight in the dark at 4°C for 20–23 h. Directly after opening the evacuated tubes, the sum of L-ascorbic acid + dehydro-L-ascorbic acid (vitamin C) was assessed in wholeblood by HPLC with fluorometric detection (Speek *et al.*, 1984). All-*trans* retinol, α-tocopherol, β-carotene and total carotenoids were assayed in plasma (stored at − 80°C) by HPLC with colorimetric detection (van Vliet *et al.*, 1991). Plasma cotinine levels were determined by gas chromatography (Feyerabend *et al.*, 1984).

### *Data analysis*

Initial baseline values and changes in these values during the intervention period were compared between the placebo group and the β-carotene group using the unpaired Student's *t*-test. Univariate log-linear Poisson regression was used to compare micronuclei counts between both groups, and to evaluate associations between micronuclei counts and other parameters. Percentual changes in micronuclei counts during the intervention trial were quantified by analysing the final/initial micronuclei counts ratio in binomial regression. Multiple Poisson regression was used to quantify the difference in micronuclei between the placebo and β-carotene groups after correction for incomplete randomisation. All data analysis were performed using the BMDP and GENSTAT packages (Dixon, 1988; GENSTAT 5 Committee, 1987).

### Results

Table I shows that the placebo and β-carotene groups are comparable for all characteristics and that, except for plasma β-carotene, only minor changes occurred during the intervention trial. In accordance with the stable cotinine levels, only one smoker (placebo group) reported to have changed his smoking habits during the trial. His plasma cotinine levels, however, hardly changed (349 and 313 nmol $ml^{-1}$ respectively). The mean body mass index (BMI) was similar in the placebo group (24.3 ± 3.0 kg $m^{-2}$) and the β-carotene group (24.7 ± 3.3 kg $m^{-2}$), and all participants but one reported stable weights during the trial (in one placebo participant BMI decreased from 24.9 ± 22.2 kg $m^{-2}$). Reported alcohol consumption was also similar in the placebo (13.4 g $d^{-1}$) and the β-carotene group (12.6 g $d^{-1}$). After 7 weeks, mean plasma β-carotene had increased 13-fold to 4.13 ± 1.79 μmol $l^{-1}$ in the supplemented group (0.26 ± 0.15 μmol $l^{-1}$ in the placebo group), and remained stable up to the end of the trial. The minimum increase in plasma β-carotene after supplementation was 1.7 fold (0.44 → 0.74 μmol $l^{-1}$), and all but four supplemented subjects had after treatment plasma levels above 1.0 μmol $l^{-1}$. Thirteen participants (12 β-carotene and, surprisingly, one placebo) reported to have observed skin yellowing during the trial, whereas one of the investigators, unaware of intervention status, noted 19 cases of skin yellowing (all β-carotene subjects). Pill counts showed that 92% of all capsules were taken (data for 103 subjects); all but four participants took more than 75% of their capsules.

At baseline, the micronuclei counts were significantly higher in the β-carotene group than in the placebo group (Figure 1 and Table II). After the intervention, however, the micronuclei counts were significantly lower in the β-carotene group (Table II). The β-carotene group thus showed a strong decrease in micronuclei counts, whereas the placebo group showed a minor, non-significant decrease (Figure 2 and Table II). In the treatment group the decrease in micronuclei was similar (47%) in subjects with final β-carotene levels above and below the median of 4.1 μmol $l^{-1}$. To obtain an unbiased estimate of the intervention effect, we calculated the difference between the placebo group and the β-carotene groups after intervention, allowing for the differences that existed between both groups before the intervention. After adjustment for initial micronuclei counts, the final micronuclei counts were estimated to be 27% lower in the β-carotene group than in the placebo group (95% CI: 9%–41%). Adjustment for the baseline characteristics given in Table I did not alter this estimate, since no associations were detected between micronuclei counts at baseline and any of the baseline characteristics listed in Table I, or alcohol consumption (All Pearson $r < 0.14$).

The micronuclei counts before and after the trial were clearly associated ($P < 0.001$), but we observed only a modest correlation in both the placebo group (Pearson $r = 0.29$) and the β-carotene group (Pearson $r = 0.40$). The micronuclei counts thus show a large within-person variation (see also Figure 2).

### Discussion

This trial in heavy smokers shows a reduction in frequency of micronucleated sputum cells after supplementation with β-carotene, suggesting that the inverse epidemiological association between β-carotene and lung cancer (Willett & MacMahon, 1984; Colditz *et al.*, 1987; Fontham, 1990) is indeed due to β-carotene, and not to associated food or life-style

**Table I** Baseline characteristics (mean ± s.d.) and changes in these characteristics during a 14-week intervention trial in male smokers, assigned to either β-carotene or placebo treatment

| | *Placebo group (n = 61)* | | *β-carotene group (n = 53)* | |
|---|---|---|---|---|
| | *Baseline values* | *Change (after-before)* | *Baseline values* | *Change (after-before)* |
| Age (yrs) | 40.0 ± 10.1 | n.a. | 40.2 ± 9.1 | n.a. |
| Number of cigarettes per day | 20.8 ± 6.7 | n.a. | 21.7 ± 6.4 | n.a. |
| Years of smoking | 21.9 ± 10.5 | n.a. | 22.0 ± 9.1 | n.a. |
| Blood vitamin C (μmol $l^{-1}$) | 37.6 ± 18.8 | − 0.4 ± 16.7 | 38.2 ± 17.2 | − 1.1 ± 16.0 |
| Plasma retinol (μmol $l^{-1}$) | 2.33 ± 0.60 | − 0.06 ± 0.42 | 2.38 ± 0.55 | − 0.01 ± 0.41 |
| Plasma α-tocopherol (μmol $l^{-1}$) | 30.5 ± 7.1 | 1.4 ± 4.3 | 31.2 ± 7.0 | 0.5 ± 3.2 |
| Plasma β-carotene (μmol $l^{-1}$) | 0.28 ± 0.18 | − 0.02 ± 0.13 | 0.32 ± 0.16 | 3.79 ± 2.02[a] |
| Plasma total carotenoids (μmol $l^{-1}$) | 1.56 ± 0.59 | 0.09 ± 0.43 | 1.49 ± 0.58 | 3.66 ± 1.93[a] |
| Plasma cotinine (ng $ml^{-1}$) | 323.1 ± 122.6 | − 8.4 ± 72.8 | 332.8 ± 109.6 | − 8.0 ± 74.1 |

[a]β-carotene group significantly different from the placebo group, $P < 0.0001$. n.a. = not applicable.

**Figure 1** Distribution of micronuclei-counts at the start of a 14-week trial in male smokers, assigned to either placebo ($n = 61$) or β-carotene ($n = 53$) treatment.

**Table II** Micronuclei counts (mean per 3,000 cells ± s.d.) and changes in micronuclei counts during a 14-week intervention trial in male smokers, assigned to either β-carotene or placebo treatment

| | *Placebo group (n = 61)* | *β-carotene group (n = 53)* |
|---|---|---|
| Micronuclei at baseline[a] | 4.0 ± 3.5 | 5.0 ± 3.4 |
| Micronuclei after 14 weeks treatment[a] | 3.4 ± 3.3 | 2.6 ± 2.8 |
| Change in micronuclei (after-before)[a] | – 0.6 ± 4.0 | – 2.3 ± 3.4 |
| % Change in micronuclei (95% confidence interval) | – 16 (– 31%– + 1%) | – 47 (– 57%– – 35%) |

[a]β-carotene group significantly different from placebo group; $P < 0.05$.

factors. These results thus support a protective role for β-carotene in the development of human cancer, as proposed by Peto *et al.* (1981). Moreover, the results indicate that β-carotene is protective in man by preventing DNA damage in target tissues, thus providing a plausible mechanism of action.

The approximately 30% reduction in micronuclei after β-carotene treatment is in accordance with the effect of β-carotene reported in buccal mucosa of betel nut chewers (Stich *et al.*, 1984*a*) and tobacco chewers (Stich *et al.*, 1985, 1988). Our findings extend these observations to cigarette smoke-induced tracheobronchial micronuclei, which may reflect lung cancer risk (Stich & Rosin, 1984*b*; Lippman *et al.*, 1990*a*,*b*). Moreover, as plasma retinol levels were not changed, this study shows that the provitamin β-carotene does not exert its action after intestinal or hepatic conversion to retinol. It thus seems that β-carotene *per se* is effective at the cellular level. The protective action of β-carotene may be explained by its anti-oxidant capacity to quench highly reactive singlet oxygen and free radical species (Krinsky, 1989). Free radicals are abundant in cigarette smoke and tar (Pryor *et al.*, 1983) and are believed to initiate cancer by damaging DNA (Pryor, 1987). In addition, β-carotene has been hypothesised to be effective after conversion to retinol at a tissue or cellular level (de Vet, 1989). β-carotene could thus rapidly compensate for local deficiencies in retinol, which may be induced by carcinogens (Edes *et al.*, 1991).

Micronuclei in exfoliated epithelial cells reflect the extent of chromosome breakage due to carcinogenic exposure, when the cells were dividing a few days or weeks earlier, in the basal layer of the epithelium of the tracheobronchial tree (Stich & Rosin, 1984*b*). As DNA damage is considered crucial in carcinogenesis (Weinstein, 1988), the frequency of micronuclei may thus reflect cancer risk. In several experimental models, including the rat bronchial carcinoma model (Stich & Rosinn, 1984*b*; Lippman *et al.*, 1990*b*), high frequencies of micronuclei are observed after carcinogen exposure. In man, numbers of micronuclei in buccal mucosa cells have been found to increase after exposure to tobacco and alcohol (Stich & Rosin, 1983*a*), betel quid (Stich *et al.*, 1982) and X-radiation (Stich *et al.*, 1983*b*); all these exposures are known causes of oral cancer. Similarly, smokers have elevated frequencies of micronucleated cells in expectorated sputum (Fontham, 1990) and bronchial brushings (Lippman *et al.*, 1990*a*). These observations strongly suggest that micronuclei indeed reflect early or intermediate stages of the carcinogenic process. Follow-up studies on the predictive value of micronuclei for cancer development, however, have not been published.

Surprisingly, our data show higher initial micronuclei counts in the β-carotene group, indicating unsuccessful randomisation. The number of inevaluable volunteers, as well as the reasons for inevaluability were similar in the placebo and the β-carotene groups, and can therefore not explain this difference. During the trial, the β-carotene and placebo groups were equally represented in all staining and scoring runs, so that any systematic difference in staining or scoring procedures between the two groups seems improbable. Moreover, all slides were coded, and scored by a single technician, and all other baseline characteristics measured (Table I) were comparable between the two groups. Regression to the mean may have influenced the observed reduction in the β-carotene group, but cannot have influenced after-treatment micro-

**Figure 2** Changes in micronuclei-counts during a 14-week trial in male smokers, assigned to either placebo ($n = 61$) or β-carotene ($n = 53$) treatment.

nuclei counts. Despite the higher initial count, the after treatment counts were significantly lower in the β-carotene group, even without adjustment for initial counts.

In the treatment group, we did not observe a dose-response relationship between plasma β-carotene and reduction in micronuclei count. However, we evaluated the effect of only one high dose of β-carotene, and almost all subjects showed dramatic increases in plasma levels. Furthermore, the limited number of subjects, the low frequency of micronuclei, as well as the considerable within-person variation in micronuclei counts make it difficult to evaluate a dose-response relationship. The low frequency of micronuclei in this study may be partly due to our stringent scoring criteria, aimed at identifying micronuclei reproducibly and with a high certainty. The large within-person variability may be explained by an inherent variability in sampling site, as expectorated cells may originate from all sites in the tracheobronchial tree. This large random sampling variation implies that the statistical power of studies using sputum is only sufficient to demonstrate large effects in study groups of considerable size. For future studies, repeated sampling and scoring can be used to diminish within-person variation. In addition, a run-in period prior to treatment could be used to assess eligibility with respect to sputum production and to stratify the treatment groups on micronuclei counts. Alternatively, studies using bronchial brushings, though more invasive, have the merit of being site-specific and may prove more useful to evaluate smaller effects, such as dose responses. Such studies may also be used to evaluate variations in counts between different sites. In addition, cellular levels of β-carotene could be studied in future studies, since plasma β-carotene levels may not wholly reflect tissue levels of β-carotene in the tracheobroncheal tree.

Our data suggest that β-carotene is effective by preventing DNA damage and may thus affect early or intermediate stages of carcinogenesis. This is in line with laboratory studies that indicate a role of β-carotene in antimutagenesis and prevention of malignant transformation (Krinsky, 1989). However, there are also indications that β-carotene may affect later stages of carcinogenesis (Prabhala *et al.*, 1991). The recently reported lack of effect of β-carotene in trials on cervical dysplasia (de Vet *et al.*, 1991) and second skin cancers (Greenberg *et al.*, 1990) may, apart from site specificity, be explained if β-carotene is primarily effective in earlier stages of carcinogenesis. To address this question, the ongoing intervention studies in cancer incidence will need a long follow-up. Indeed, the β-carotene trial in the Physicians Health Study has recently been extended to cover more than 10 years follow-up (Manson *et al.*, 1991).

This study yields evidence that the observed inverse association between β-carotene and lung cancer is due to β-carotene *per se*. Though the predictive value of micronuclei for cancer risk remains to be shown definitively, our results suggest that β-carotene may indeed reduce human cancer risk. It is clear that the health benefits of stopping smoking will far outweigh those of dietary changes (Colditz *et al.*, 1987). These results should therefore not be explained as a way to prevent lung cancer in people who continue to smoke.

We thank the employees and companies who participated in this study: Dr M.J. Jarvis (Institute of Psychiatry, London) for the cotinine assays; Dr J. Schrijver for the vitamin assays; Ms T. Bruyntjes-Rozier for scoring micronuclei; E.D. Schoen MSc for statistical advice; W.J.M.J. Gorgels MD, Ms W. Stenhuis and Ms H. Leezer-de Hoog for assistance in data collection. β-carotene and placebo capsules were generously provided by F. Hoffman-La Roche Ltd. This study was financially supported by the Dutch Prevention Fund and TNO Nutrition and Food Research.

### References

ALBANES, D., VIRTAMO, J., RAUTALAHTI, M., PIKKARAINEN, J., TAYLOR, P.R., GREENWALD, P. & HEINONEN, O.P. (1986). Pilot study: the US-Finland lung cancer prevention trial. *J. Nutr. Growth Cancer*, **3**, 207–214.

BRUCK, H.C. (1981). *Histologische Technik. Leitfaden für die Herstellung mikroskopischer Präparate in Unterricht und Praxis.* ISBN 3-13314304-2. Georg Thieme Verlag, Stuttgart New York.

COLDITZ, G.A., STAMPFER, M.J. & WILLETT, W.C. (1987). Diet and lung cancer – a review of the epidemiological evidence in humans. *Arch. Intern. Med.*, **147**, 157–160.

DE VET, H.C.W. (1989). The puzzling role of vitamin A in cancer prevention (review). *Anticancer Res.*, **9**, 145–152.

DE VET, H.C.W., KNIPSCHILD, P.G., WILLEBRAND, D., SCHOUTEN, H.G.A. & STURMANS, F. (1991). The effect of beta-carotene on the regression and progression of cervical dysplasia: a clinical experiment. *J. Clin. Epidemiol.*, **44**, 273–283.

DIXON, W.J. (1988) (ed). *BMDP Statistical Software Manual.* University of California Press, Berkeley.

EDES, T.E., GYSBERS, D.G., BUCKLEY, C.S. & THORNTON, W.H. (1991). Exposure to the carcinogen benzopyrene depletes tissue vitamin A: β-carotene prevents depletion. *Nutr. Cancer*, **15**, 159–166.

EDITORIAL (1991). A carrot a day keeps cancer at bay? *Lancet*, **337**, 81–82.

FEYERABEND, C., BRYANT, A., JARVIS, M.J. & RUSSELL, M.A.H. (1984). Determination of cotinine in biological fluids of non-smokers by packed column gas-liquid chromatography. *J. Pharm. Pharmacol.*, **38**, 1075–1078.

FONTHAM, E.T., CORREA, P., RODRIGUEZ, E. & LIN, Y. (1986). Validation of smoking history with the micronuclei test. In *Mechanisms in Tobacco Carcinogenesis*, Hoffmann, D. & Harris, C.C. (eds): Banbury Report 23, 113–118, Cold Spring Harbor Laboratory, Cold Spring Harbor, New York.

FONTHAM, E.T. (1990). Protective dietary factors and lung cancer. *Int. J. Epidemiol.*, **19** (suppl 1), S32–S42.

GENSTAT 5 COMMITTEE (1987). *GENSTAT 5 Reference Manual.* Oxford University Press: Oxford.

GREENBERG, E.R., BARON, J.A., STUKEL, T.A., STEVENS, M.M., MANDEL, J.S., SPENCER, S.K., ELIAS, P.M., LOWE, N., NIERENBERG, D.W., BAYRD, G., VANCE, J.C., FREEMAN, D.H., CLENDENNING, W.E., KWAN, T. & THE SKIN CANCER PREVENTION STUDY GROUP. (1990). A clinical trial of beta-carotene to prevent basal-cell and squamous-cell cancers of the skin. *N. Engl. J. Med.*, **323**, 789–795.

HENNEKENS, C.H. & EBERLEIN, K. (1985). A randomized trial of aspirin and β-carotene among US physicians. *Prev. Med.*, **14**, 165–168.

KRINSKY, N.I. (1989). Carotenoids as chemopreventive agents. *Prev. Med.*, **18**, 592–602.

LIPPMAN, S.M., PETERS, E.J., WARGOVICH, M.J., STADNYK, A.N., DIXON, D.O., DEKMEZIAN, R.H., LOEWY, J.W., MORICE, R.C., CUNNINGHAM, J.E. & HONG. W.K. (1990*a*). Bronchial micronuclei as a marker of an early stage of carcinogenesis in the human tracheobronchial epithelium. *Int. J. Cancer*, **45**, 811–815.

LIPPMAN, S.M., PETERS, E.J., WARGOVICH, M.J., DIXON, D.O., DEKMEZIAN, R.H., CUNNINGHAM, J.E., LOEWY, J.W., MORICE, R.C. & HONG, W.K. (1990*b*). The evaluation of micronuclei as an intermediate endpoint of bronchial carcinogenesis. *Advances in Cancer Control: Screening and Prevention Research*, pp. 165–177. Wiley-Liss, New York.

MANSON, J.E., HUNTER, D.J., BURING, J.E. & HENNEKENS, C.H. (1991). Beta carotene to prevent skin cancer. *N. Engl. J. Med.*, **324**, 924.

MEYSKENS, F.L. (1990). Coming of age – the chemoprevention of cancer. *N. Engl. J. Med.*, **323**, 825–827.

PETO, R., DOLL, R., BUCKLEY, J.D. & SPORN, M.B. (1981). Can dietary β-carotene materially reduce human cancer rates? *Nature*, **290**, 201–208.

PRABHALA, Rh., GAREWAL, H.S., HICKS, M.J., SAMPLINER, R.E. & WATSON, R.R. (1991). The effects of 13-cis-retinoic acid and beta-carotene on cellular immunity in humans. *Cancer*, **67**, 1556–1560.

PRYOR, W.A., HALES, B.J., PREMOVIC, P.I. & CHURCH, D.F. (1983). The radicals in cigarette tar: their nature and suggested physiological complications. *Science*, **220**, 425–427.

PRYOR, W.A. (1987). Cigarette smoke and the involvement of free radical reactions in chemical carcinogenesis. *Br. J. Cancer*, **55**, suppl VIII, 19–23.

SACCOMANNO, G. (1978). *Diagnostic Pulmonary Cytology.* ISBN 0-89189-050-5. American Society of Clinical Pathologists, Chicago.

SPEEK, A.J., SCHRIJVER, J. & SCHREURS, W.H.P. (1984). Fluorimetric determination of total vitamin C in whole blood by high performance liquid chromatography with pre-column derivatization. *J. Chromatogr.*, **305**, 53–60.

STICH, H.F., STICH, W. & PARIDA, B.B. (1982). Elevated frequency of micronucleated cells in the buccal mucosa of individuals at high risk for oral cancer: betel quid chewers. *Cancer Lett.*, **17**, 125–134.

STICH, H.F. & ROSIN, M.P. (1983*a*). Quantitating the synergistic effect of smoking and alcohol consumption with the micronucleus test on human buccal mucosal cells. *Int. J. Cancer*, **31**, 305–308.

STICH, H.F., SAN, R.H.C. & ROSIN, M.P. (1983*b*). Adaptation of the DNA-repair and micronucleus test on human cell suspensions and exfoliated cells. *Ann. NY Acad. Sci.*, **407**, 93–105.

STICH, H.F., ROSIN, M.P. & VALLEJERA, M.O. (1984*a*). Reduction with vitamin A and beta-carotene administration of proportion of micronucleated buccal mucosal cells in asian betel nut and tobacco chewers. *Lancet*, **i**, 1204–1206.

STICH, H.F. & ROSIN, M.P. (1984*b*). Micronuclei in exfoliated human cells as a tool for studies in cancer risk and cancer intervention. *Cancer Lett.*, **22**, 241–253.

STICH, H.F., HORNBY, P. & DUNN, B.C. (1985). A pilot β-carotene intervention trial with Inuits using smokeless tobacco. *Int. J. Cancer*, **36**, 321–327.

STICH, H.F., ROSIN, M.P., HORNBY, A.P., MATTHEW, B., SANKARANARAYANAN, R. & NAIR, M.K. (1988). Remission of oral leukoplakias and micronuclei in tobacco/betel quid chewers treated with beta-carotene and with beta carotene plus vitamin A. *Int. J. Cancer*, **42**, 195–199.

VAN VLIET, T., VAN SCHAIK, F., VAN SCHOONHOVEN, J. & SCHRIJVER, J. (1991). Determination of several retinoids, carotenoids, and E vitamers by HPLC. Application to plasma and tissues of rats fed a diet rich in either β-carotene or canthaxanthin. *J. Chromatogr.*, **553**, 179–186.

WEINSTEIN, I.B. (1988). The origins of human cancer: molecular mechanisms and their implications for cancer prevention and treatment. *Cancer Res.*, **48**, 4135–4143.

WILLETT, W.C. & MACMAHON, B. (1984). Diet and cancer – an overview. *N. Engl. J. Med.*, **310**, 633–638.